# Headaches in Healthcare Workers: A Prospective Study of Precipitating and Maintenance Variables and Their Relationship with Burnout as a Post-COVID Syndrome

**DOI:** 10.3390/neurolint16060109

**Published:** 2024-11-14

**Authors:** Fernanda Gil-Almagro, Francisco Javier Carmona-Monge, Fernando José García-Hedrera, Cecilia Peñacoba-Puente

**Affiliations:** 1 Department of Psychology, Faculty of Health Sciences, Rey Juan Carlos University, Avda. de Atenas, s/n, 28922 Madrid, Spain; fgilalmagro@gmail.com; 2 Intensive Care Unit, Hospital Universitario Fundación Alcorcón, Calle Berlin, 6A, 28922 Madrid, Spain; fjgarciah@gmail.com; 3 Department of Anaesthesiology and Reanimation, Hospital Universitario Santiago de Compostela, Rúa Choupana s/n, 15706 A Coruña, Spain; javichun@gmail.com

**Keywords:** headache, health care workers, post-pandemic, cognitive fusion, burnout

## Abstract

Background: Headaches are a common symptom in healthcare workers (HCWs), mainly associated with high levels of stress. Different research has studied their incidence during the COVID-19 pandemic, most of them with correlational designs, and at the beginning of the pandemic and focused on the associated occupational variables. Aims: (1) To analyze the incidence of headaches in HCWs at the beginning of the COVID-19 pandemic and their maintenance six months later. (2) To explore the risk factors associated with their onset and maintenance, including sociodemographic, occupational, emotional symptomatology, and personality variables. (3) To propose a model to explain the chronification of stress in burnout, including the moderating role of chronic headaches. Methods: A prospective study (*n* = 259 HCWs) at three points in time during the COVID-19 pandemic, from the alarm state phase (T1: May–June 2020) to the post-pandemic stage (T3: April–July 2022), including an intermediate measure six months after T1 (T2). Descriptive analyses, Pearson’s chi-square, Student’s t, logistic regressions, and moderated mediation models were conducted using the Process package for SPSS. In addition to headaches, socio-demographic, occupational, emotional symptomatology, and personality variables were included. Results: At T1 the prevalence of headaches was 69.9%. At T2 the prevalence was 73.7%. Of these, 59.5% are T1–T2 sustained headaches. Headaches at T1 were associated with age (*p* = 0.010) (younger HCWs), professional category (*p* = 0.049) (nurses), service (*p* = 0.023) (ICU, COVID hospitalization), non-availability of PPE (*p* = 0.010), additional COVID-19 symptomatology (*p* < 0.001), and concern for contagion of family members (*p* < 0.001) (higher scores). In addition, HCWs with headaches had higher levels of stress (*p* = 0.001), anxiety (*p* = 0.001), depression (*p* = 0.041), and sleep disorders (*p* < 0.001). A subsequent logistic regression analysis showed that of the above variables, the presence of additional COVID-19 symptoms (*p* < 0.001) and depression (*p* = 0.010) were the predictor variables. With regard to the maintenance of headaches (T1–T2), anxiety (*p* = 0.035), stress (*p* = 0.001), and cognitive fusion (*p* = 0.013) were found to be the significant variables. The tested model proposes anxiety (T1) as antecedent, cognitive fusion (T2) as mediator, burnout (T3) as consequent, and chronic headaches (yes/no) as the moderating variable between anxiety and burnout (model 5). The model is significant (F = 19.84, *p* < 0.001) and contributes to the explanation of 36% of the variance of burnout. The relationships in the model are all statistically significant, and specifically chronic headaches contribute to a 6-fold increase in the likelihood of burnout. Conclusions: The present research differentiates between precipitating and maintenance factors of headaches in HCWs. The former, more studied in previous research, are usually related to sociodemographic and occupational variables and levels of anxiety and stress. Maintenance factors, scarcely explored, are related to the maintenance of emotional symptomatology and the inability to manage intrusive thoughts (i.e., cognitive fusion). Of particular interest is that the presence of chronic headaches itself is capable of producing burnout as a post-COVID syndrome.

## 1. Introduction

Headaches are a high prevalence disorder in the general population, being among the most prevalent conditions in the world. In Europe, the prevalence in different studies ranges from 53% to 75% of the population suffering from different types of headache disorders [1,2]. Healthcare workers (HCWs) are a subgroup of the population that has been widely studied, as the prevalence of headaches in them is more elevated than in the general population, with studies indicating values as high as 88% prevalence and an increased predominance in women [3].

Biological factors also contribute to the high prevalence of headaches among HCWs, with genetic predisposition being a notable component. Research has shown that specific genetic variations in neurotransmitter and ion channel regulation are associated with an increased susceptibility to migraine, which could explain why certain individuals experience headaches more frequently in response to common triggers found in healthcare settings, such as bright lighting and stress [4]. This genetic sensitivity, combined with the occupational demands of healthcare work, may contribute to the elevated headache rates observed in HCWs compared to the general population.

There are different situations identified as risk factors in the literature for the development of headaches in HCWs. Perceived stress is one of the highlighted factors as it has been shown to increase the rate of headaches in nurses. Increased workload has also shown a relationship with increased mental fatigue, neck pain, and headaches [5]. Shift rotations and working night shifts has also been identified as an important source of headaches in HCWs [6,7]. In addition, hormonal factors, particularly in female healthcare professionals, further compound headache prevalence. Hormonal fluctuations related to the menstrual cycle, pregnancy, and menopause are known to increase headache frequency and intensity. This predisposition is often exacerbated by the irregular schedules and disrupted sleep patterns common in healthcare work, which can disturb circadian rhythms and increase headache susceptibility [8,9].

In a similar way, working in highly specialized care units (such as critical care units) has also been suggested as an additional risk factor for the development of headaches [10,11].

During the COVID-19 pandemic, the main protective measure for primary prevention of contagion was the use of personal protective equipment (PPE) [12]. The virus is highly contagious and is transmitted through the respiratory tract and airborne droplets. This made the use of this equipment extremely important to provide safe and effective care. This equipment included special breathing masks, face shields, glasses, gloves, and overalls. The availability of this equipment during the crisis was reduced, and the workload was extremely high, making it not possible to make pauses and remove these devices for short periods of time. All these situations lead to the development of complications such as adverse skin reactions, allergic reactions, and headaches as were reported by HCWs [13,14]. An association between the COVID-19 vaccine and headaches has also been found in several studies, indicating that up to 30% of HCWs reported post-vaccination headaches, and this association is more important in women [15,16].

Several epidemiological studies have identified various risk factors that contribute to an increased likelihood of developing chronic headaches in HCWs. Long working hours, irregular sleep patterns, high levels of stress, and the physical demands of the job can contribute to chronic headaches [1,17]. Additionally, dehydration, excessive caffeine consumption, and poor posture during prolonged shifts increase susceptibility. The constant exposure to bright lights, noise, and the need for personal protective equipment (PPE), such as tight-fitting masks, can also trigger tension headaches or migraines. Addressing these factors is crucial to improving the well-being and productivity of healthcare professionals [18]. Research has also indicated that the chronic exposure to high stress, typical of healthcare environments, leads to sustained elevated cortisol levels. This hormonal imbalance has been shown to influence pain pathways, increasing the sensitivity to headache triggers and contributing to long-term changes in headache patterns among those with chronic exposure to occupational stress [19,20].

Mental disorders, such as anxiety, depression, and insomnia, have a significant impact on the frequency and severity of headaches. These conditions can increase stress levels and lead to tension, which often triggers or exacerbates headaches. Anxiety and depression are also associated with increased muscle tension and altered pain perception, making individuals more sensitive to headache pain. Furthermore, sleep disturbances caused by insomnia can worsen both the frequency and intensity of headaches [21]. Research shows that headaches can significantly impact a person’s life, affecting their work performance, finances, sexual health, mental well-being, social interactions, and emotional stability [22,23]. Moreover, headaches are strongly linked to reduced workplace attendance, ability, and productivity. As a result, the World Health Organization (WHO) recognizes headaches as a major cause of disability [24,25].

It is therefore of particular interest to further investigate the variables linked to the development and maintenance of headaches in HCWs. Based on this need, the present research is carried out through a longitudinal design that includes from the beginning of the pandemic until two years later, analyzing the maintenance of headaches in the so-called post-pandemic stage. In addition to sociodemographic and occupational variables, emotional symptomatology and psychosocial variables will be included, adopting a person–environment interactionist perspective, especially useful in preventive programs. Specifically, the aim of this research was to analyze the incidence of headaches in HCWs at the start of the COVID-19 pandemic and assess how many developed chronic headaches over six months. Additionally, we explored socio-demographic, occupational, and psychosocial factors linked to the onset of headaches and personality traits and psychosocial factors associated with the persistence of headaches over time. We propose a model to explain the chronification of anxiety in HCWs, using burnout syndrome as a post-COVID-19 example, while assessing the role of chronic headaches in this model.

## 2. Materials and Methods

### 2.1. Design

This study employed a prospective longitudinal design, collecting data across three distinct time points: (1) from 1 May to 21 June 2020, corresponding to the final phase of the state of alarm implemented in Spain on 14 March, which included a lockdown until 21 June 2020; (2) approximately six months after the cessation of the state of alarm, from January to April 2021, when the pandemic situation in Spain remained severe, with 3,347,512 confirmed cases and 76,328 deaths recorded by 9 April 2021; and (3) one year following the second assessment period, from April to July 2022. By this third phase, confirmed COVID-19 cases in Spain had escalated to 12,973,615, with 108,730 deaths. Participants’ headache experiences were assessed during the first and second evaluation points to monitor for potential chronification over time.

**Figure 1:**
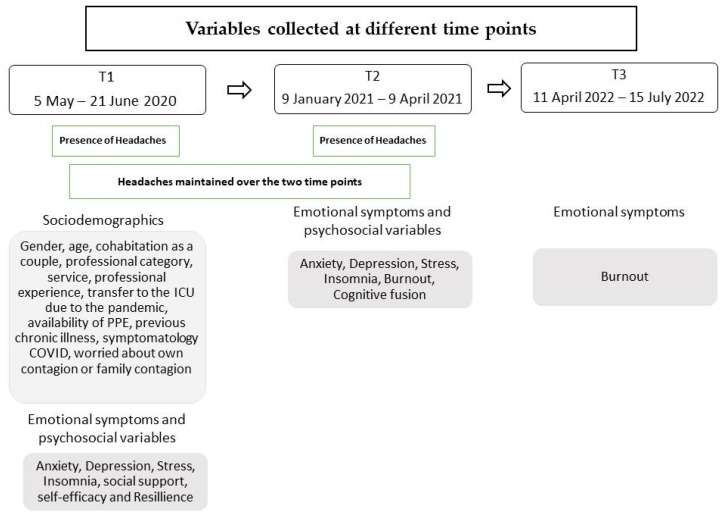
Variables assessed at each of the time points of the study (T1, T2, T3).

Additionally, various sociodemographic, occupational, and psychosocial variables were measured during the first, second, and third time points (refer to the Instruments section for more detailed information). Figure 1 outlines the evaluation periods and the variables involved in the research.

### 2.2. Procedure and Participants

Data were collected through an online electronic survey specifically developed by the research team for this study. At the outset of the questionnaire, participants were briefed on the primary objectives of the study and were requested to give informed consent. This consent included explicit authorization to use their email addresses for follow-up communications in subsequent evaluation phases.

The sample consisted of HCWs from various departments of the Spanish National Healthcare System. A probabilistic convenience sampling approach was utilized, applying the following inclusion criteria: being a HCW (physician, nurse, or technician), actively employed within the Spanish National Healthcare System (across both public and private sectors), aged 18 years or older, and having direct contact with COVID-19 patients. The exclusion criterion was restricted to HCWs who had been employed across multiple departments during the data collection period. An initial reference sample size of *n* = 120 was established for prospective studies [26]. However, considering the expected decrease in participation rates associated with the longitudinal design of the study and the difficult circumstances encountered by HCWs during the COVID-19 pandemic [27,28], a target sample size of at least 720 participants was established for the first data collection point. Ultimately, 1121 HCWs took part in the first evaluation phase. Out of these, 403 continued to participate in the second phase, and 259 remained involved by the third evaluation phase, forming the final sample size of the study (*n* = 259).

To recruit participants, a link to the questionnaire was sent to HCWs within the Spanish National Healthcare System, both public and private. The questionnaire was distributed via social media platforms (Facebook, LinkedIn, Twitter, and WhatsApp) as well as through official email channels within public and private healthcare services in Spain. For the second and third phases, follow-up emails were sent to HCWs who had participated in the initial evaluation, inviting them to take part in the subsequent stages of the study.

### 2.3. Variables and Instruments

#### 2.3.1. Presence of Headache [T1 and T2]

Headaches were evaluated as part of a broader set of symptoms related to COVID-19. Specifically, a custom self-report questionnaire created by the research team was used. The questionnaire listed 13 symptoms, including headaches, as well as fever, chills, cough, muscle pain, shortness of breath, dizziness, rhinitis, sore throat, chest pain, loss of smell (anosmia), loss of taste (ageusia), and skin manifestations. The questionnaire began with the following question: “Have you experienced any of the following COVID-19-related physical symptoms in the past few weeks?” Participants responded to each symptom (including headaches) with a yes or no answer.

#### 2.3.2. Sociodemographic and Occupational Variables [T1]

Sociodemographic variables (age, gender, cohabitation as a couple, previous chronic illness) and occupational variables (professional category, service, professional experience, transfer to the ICU due to the pandemic, availability of PPE) were collected using an ad hoc questionnaire designed by the research team.

To assess concerns regarding COVID-19, two ad hoc items were developed: one evaluating concern about personal infection and the other addressing concern about infecting a family member. Both items utilized a 4-point Likert-type scale, ranging from 1 (“not at all concerned”) to 4 (“very concerned”).

#### 2.3.3. Variables Related to Symptoms

(a) Depression, Anxiety and Stress: [T1 and T2]

To assess symptoms of anxiety, depression, and stress, the Spanish version of the Depression, Anxiety, and Stress Scale (DASS-21) [29,30] was administered. Each scale contains seven items with a 4-point Likert response format, ranging from 0 (“did not apply to me”) to 3 (“applied to me a lot” or “most of the time”). Scores for each dimension can range from 0 to 21 points. In this study, Cronbach’s alpha values were 0.81, 0.84, and 0.89 for depression, anxiety, and stress, respectively, at T1, and 0.83, 0.87, and 0.84 for depression, anxiety, and stress, respectively, at T2.

(b) Insomnia [T1 and T2]

Insomnia symptoms were assessed using the Spanish version of the Insomnia Severity Index (ISI) [31,32]. This instrument offers a concise evaluation of insomnia symptoms, aligning with the criteria from the “Diagnostic and Statistical Manual of Mental Disorders” and the “International Classification of Sleep Disorders”. The ISI consists of seven items that measure three main components: severity, impact, and satisfaction. Responses are recorded on a Likert scale ranging from 0 (“no problem”) to 4 (“severe problem”), resulting in a total score between 0 and 28. A score of 22 or above indicates severe clinical insomnia. In this study, the Cronbach’s alpha for the scale was 0.87 at T1 and 0.85 at T2.

#### 2.3.4. Psychosocial and Personality Variables

(a) Social support [T1]

Social support was assessed using the Spanish version [33] of the Multidimensional Scale of Perceived Social Support (MSPSS) [34]. This instrument comprises 12 items across three dimensions: family, friends, and significant others, with four items per dimension. Responses are scored on a 7-point Likert scale, ranging from 1 (“completely disagree”) to 7 (“completely agree”). A total social support score is derived by summing the three subscale scores. The MSPSS has demonstrated strong psychometric properties across various studies. In this study, Cronbach’s alpha for overall social support was 0.85, with high internal consistency for the dimensions: family support (0.81), friend support (0.82), and support from significant others (0.79).

(b) Self-efficacy [T1]

The General Self-Efficacy Scale (GSES) [35] in its Spanish adaptation was employed to assess self-efficacy [36]. This instrument consists of 10 items rated on a 4-point Likert scale, where responses range from 1 (“completely disagree”) to 4 (“completely agree”), yielding a total possible score between 10 and 40. In the context of our study, the GSES demonstrated excellent internal consistency, with a Cronbach’s alpha coefficient of 0.91.

(c) Resilience [T1]

The Resilience Scale (RS-14), in its Spanish version, was utilized for resilience assessment [37]. This scale comprises 14 items, each evaluated on a 7-point Likert scale, ranging from 1 (“strongly disagree”) to 7 (“strongly agree”). The total possible score spans from 14 to 98, with higher scores indicating greater resilience levels. In our study, the RS-14 demonstrated strong internal consistency, with a Cronbach’s alpha of 0.94.

(d) Cognitive fusion [T2]:

For assessing cognitive fusion, the Spanish version of the Cognitive Fusion Questionnaire (CFQ) [38] was applied [39]. This instrument comprises seven items designed to evaluate the degree to which individuals are psychologically influenced or governed by their thoughts. Responses are recorded on a 7-point Likert scale, from 1 (“never”) to 7 (“always”), with total scores ranging from 7 to 49. Higher scores suggest a greater degree of cognitive fusion. In our study, the CFQ demonstrated excellent internal consistency, with a Cronbach’s alpha of 0.97.

(e) Burnout [T3]:

The Maslach Burnout Inventory-Human Services Survey (MBI-HSS) was used [40] in its Spanish version [41]. It consists of 22 items that adopt a 7-point Likert-type response format, ranging from 0 (never) to 6 (every day). The instrument assesses three dimensions or subscales of burnout: emotional exhaustion, depersonalization and reduced personal accomplishment. In our study, we considered the total burnout. The Cronbach’s alpha was 0.87.

The Spanish adaptation of the Maslach Burnout Inventory-Human Services Survey (MBI-HSS) [40] was utilized for burnout assessment [41]. This tool includes 22 items, each rated on a 7-point Likert scale from 0 (“never”) to 6 (“every day”). The MBI-HSS evaluates three distinct dimensions of burnout: emotional exhaustion, depersonalization, and reduced personal accomplishment. In this study, we analyzed overall burnout. The instrument demonstrated strong internal consistency, with a Cronbach’s alpha of 0.87.

### 2.4. Data Analysis

The SPSS Statistics software, version 22 (IBM Corp., Armonk, NY, USA), was employed for data analysis. Descriptive statistics and Cronbach’s alpha reliability assessments were conducted. Qualitative variables were summarized using frequencies (*n*) and percentages (%), while quantitative variables were described with means (M) and standard deviations (SD). To examine bivariate associations and assess potential covariates, Student’s *t*-test and Pearson’s chi-square test were applied, contingent on the type of variables under analysis. To analyze the variables associated with both the presence of headaches at T1 and sustained headaches (T1 and T2), we performed two binary linear regression analyses with headaches as the dependent variable and sociodemographic, occupational variables, emotional symptomatology, and personality variables as independent variables. Only those variables that were significant in the bivariate analyses (Student’s *t*-test and Pearson’s chi-square) were included in the analyses. Finally, for the moderated mediation analysis, SPSS macro PROCESS (model 5) version 3.5 (IBM, Armonk, NY, USA) was used. A model of anxiety (T1) as antecedent, cognitive fusion (T2) as mediator and burnout syndrome (T3) as consequent is proposed. The presence of chronic headaches is proposed as a moderating variable between anxiety and burnout syndrome. Age and professional category (nurse vs. other professions) were entered as covariates. The regression/trajectory coefficients are all in non-standardized form since the standardized coefficients generally do not have a useful substantive interpretation. The model fit was also examined using the following criteria: a chi-square/df of ≤2, a *p* value of >0.05, a comparative fit index of ≥0.95, and an approximation of the mean squared error of <0.06. We have reported risk difference (RD) with 95% confidence intervals (CI). The level of significance was set to *p* < 0.05.

## 3. Results

### 3.1. Characteristics of the Sample

Table 1 presents the sociodemographic and occupational characteristics of the participants. The sample consisted of 259 HCWs, including 151 nurses (58.3%), 65 physicians (25.1%), and 43 technicians (16.6%), with an average age of 44.36 years (SD = 9.84), ranging from 21 to 66 years. The majority were female (*n* = 211, 81.5%), and most were married or in a stable relationship (*n* = 182, 70.3%). In terms of service distribution, the highest concentrations were in the ICU (*n* = 93, 35.9%) and hospitalization units (*n* = 64, 24.7%). Participants had an average of 11.06 years of professional experience (SD = 9.29), with a range from 0 to 35 years. When analyzed to be transferred to the ICU due to the pandemic, only 43 (16.6%) of the HCWs were transferred to ICU. Approximately 60% of the participants (*n* = 151) claimed lack of PPE availability at the beginning of the pandemic. Concerns about infecting a family member (Mean = 3.54, SD = 0.84) were higher than concerns about self-contagion (Mean = 2.70, SD = 0.89).

### 3.2. Prevalence of Headache and Chronic Headache

Table 2 shows the incidence of headaches at the first time point (T1; between 5 May and 21 June 2020) and at the second time point (T2; January–April 2021), as well as the incidence of sustained headaches across the two time points (T1 and T2). As can be seen in Table 2, headache cases increase slightly from T1 to T2 (from 70% to 74%). Almost 60% of the participants maintain the headaches throughout the two time points (chronic headache).

### 3.3. Relationships of Headaches (T1) with Socio-Demographic and Occupational Variables, Emotional Symptomatology and Psychosocial Variables

Table 1 shows the relationships between the incidence of headaches at the first time point (T1) and socio-demographic, occupational, emotional symptomatology, and psychosocial variables. As shown in Table 1, the following variables maintain significant results: age (*p* = 0.010) with a higher prevalence of headaches in younger HCWs, professional category (*p* = 0.049) with a higher prevalence of headaches in nurses, the service where HCWs work (*p* = 0.023) with higher prevalence of headaches in COVID hospitalization (80%), emergency (74%) and ICU (71%), the availability of PPE (*p* = 0.010) with higher prevalence of headaches in HCWs who did not have PPE (75% vs. 63%), the additional COVID-19 symptomatology (*p* < 0.001) with higher score in those with headaches (4.26 vs. 1.23), and concern for contagion of family members (*p* < 0.001) with higher score in those with headaches (3.67 vs. 3.22). In addition, HCWs with headaches had higher levels of stress (*p* = 0.001), anxiety (*p* = 0.001), depression (*p* = 0.041), and sleep disorders (*p* < 0.001). The largest effect sizes are observed for additional COVID-19 symptomatology, concern about infecting a family member, and for associated emotional symptomatology, especially for anxiety, stress, and insomnia.

### 3.4. Logistic Regression for Headache Prediction at the First Time Point

Table 3 shows the results of the binary logistic regression using the presence of T1 headaches as the dependent variable. All socio-demographic, occupational, emotional symptomatology, and personality variables that were significant in the bivariate analyses were entered as independent variables (see Table 1). As can be seen in Table 3, the presence of additional COVID-19 symptoms (*p* < 0.001) and depression (*p* = 0.010) were the predictor variables. In the set and interaction of the variables considered, additional COVID-19 symptomatology is shown to be a risk factor, increasing the probability of having a headache by 300%. A particularly surprising finding is the protective role of depression, contributing to a 21% reduction in the probability of suffering from headaches.

### 3.5. Relationships of Chronic Headaches with Emotional Symptomatology and Psychosocial Variables

With regard to aim 3, Table 4 shows personality and emotional symptomatology factors related to the maintenance of headaches (T1 and T2). As can be observed, anxiety (*p* = 0.035), stress (*p* = 0.001), and cognitive fusion (*p* = 0.013) were found to be the significant variables. In all cases, HCWs with headaches have higher scores on these variables. Similarly, HCWs with chronic headaches had higher burnout scores on T3 (*p* = 0.017). In general, the observed effect sizes are small. Moderate effect sizes are observed for stress, cognitive fusion, and burnout.

### 3.6. Logistic Regression for Chronic Headache Prediction (Only Variables Statistically Significant at the Bivariate Level Were Taken into Account

Table 5 shows the results of the binary logistic regression using the maintenance of headaches in T1 and T2 as the dependent variable. All personality and emotional symptomatology that were significant in the bivariate analyses were entered as independent variables (see Table 4). As can be seen in Table 5, stress was the predictor variable (*p* = 0.022), indicating that stress contributes to a 10.4% increase in the likelihood of chronic headaches.

### 3.7. Proposed Model to Explain the Chronification of Anxiety in Burnout Through Cognitive Fusion, Using the Presence of Chronic Headaches as a Moderating Variable

**Figure 2:**
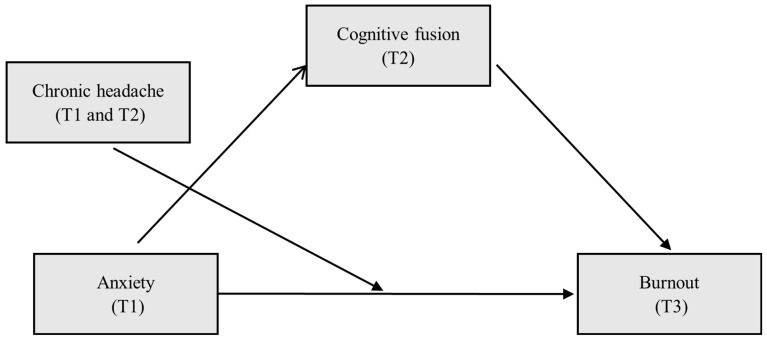
Proposed theoretical model.

Regarding the fourth aim, a model of anxiety (T1) as antecedent, cognitive fusion (T2) as mediator, and burnout syndrome (T3) as consequent is proposed. The presence of chronic headaches is proposed as a moderating variable between anxiety and burnout syndrome (model 5). Age and professional category (nurse vs. other professions) were entered as covariates (see Figure 2 and Table 6).

Table 6 shows the results found in the moderated mediation model. The model is significant (F = 19.84, *p* < 0.001) and contributes to the explanation of 36% of the variance of burnout. The relationships in the model are all statistically significant, and specifically chronic headaches contribute to a 6-fold increase in the likelihood of burnout syndrome. Significant effects of anxiety on cognitive fusion (*p* < 0.001) and of cognitive fusion on burnout (*p <* 0.001) can be observed. Similarly, direct effects of anxiety on burnout are observed (*p* < 0.001). Moderating effects of the presence of chronic headaches on the relationship between anxiety and burnout are observed. Specifically, in the absence of headaches, the relationship between anxiety and burnout is statistically significant and increases by 1.25.

## 4. Discussion

The present study aims not only to study the prevalence of headaches in health professionals during the passage of the pandemic, but also to study the possible sociodemographic, occupational, and personality variables associated with their chronification. In addition, it proposes a model that explains the effect of chronic headaches on burnout suffered in the long-term, in the so-called post-pandemic stage.

Our results showed that between 70% and 74% of HCWs experienced headaches at the onset of the COVID-19 pandemic and six months later, respectively. Sixty percent of HCWs reported persistent headaches between the two time points. These results are consistent with previous research that also reported a high prevalence of headaches among HCWs during the pandemic. A systematic review by Sahebi et al. (2022) found a high percentage of HCWs who did not report headaches prior to the pandemic but did report headache episodes during the COVID-19 pandemic [42]. Some studies linked these headaches to prolonged use of PPE, stress during the pandemic, and work overload [43]. Other authors identified headaches as one of the most prevalent symptoms in healthcare personnel and emphasized that their occurrence was closely related to physical and emotional exhaustion [44].

The chronification of headaches over time, as reported in the present study, has not been studied as much by different authors, making it necessary to delve into this line of research. Our study addresses a high percentage (59.5%) of HCWs who reported headaches at two temporal moments (chronic headache); given the lack of longitudinal studies we cannot contrast these results.

The results of the present study affirm that the development of headache at the onset of the COVID-19 pandemic was significantly related to age, being a nurse, lack of PPE availability, concern about infecting a family member, and the presence of COVID-19 symptomatology. In addition, significant correlations were observed with elevated levels of anxiety, depression, stress, and insomnia. These findings coincide with those reported by some authors who affirm that the youngest personnel have been the most affected at the beginning of the pandemic, reporting the highest levels of anxiety, depression, stress, and insomnia [45]; however, other authors claim a not very clear relationship between age and the development of headaches [46]. Previous studies affirm the lack of differentiation between professional category and the development of headaches, pointing out that physicians and nurses show similar prevalence percentages [1].

Our study follows the same line as other authors who state that the limited or inadequate use of PPE increases the risk of headaches [47]. Stress and concern about contagion from family members have also been cited as key triggers of headaches in HCWs [43,44]. Likewise, previous studies point out that psychological disorders such as anxiety, depression, and insomnia exacerbate the occurrence of headaches underscoring the need for early and preventive interventions to improve the mental well-being of HCWs [48]. However, when entering these variables in the logistic regression analyses to predict headaches, only COVID-19 symptomatology and depression are shown as predictor variables within our model. COVID-19 symptomatology is shown to be a risk factor increasing the development of headaches by 300%. These results are in line with many authors who state that COVID-19 symptoms are closely linked to headaches [49,50].

An interesting finding of our study is the protective role of depression against the onset of headaches, reducing the probability of suffering headaches by 21%. This finding contradicts most previous studies, which have shown that depression tends to be a risk factor for the chronification of headaches, especially in relation to migraine [21,51]. A recent study claims that depression significantly increases the risk of developing migraine and of migraine chronification, stating that patients with higher levels of depressive symptoms have worse prognosis in terms of frequency and severity of headache attacks [52]. Given the paucity of previous research on this subject, some considerations and hypotheses could be put forward to explain this result. Firstly, it should be noted that our bivariate analyses point to the role of depression as a risk variable for headaches, as previous research has shown [16,46]. The ‘seemingly contradictory’ result occurs in multivariate analyses, when depression, together with other psychosocial variables such as additional COVID-19 symptomatology, considering their interactions, goes on to predict headaches. Although more studies are needed, some hypotheses could be put forward. One could be a treatment effect, as people with depression may be receiving treatment that could also be helping to reduce the frequency or severity of headaches. Psychological adaptation processes could also be considered, since people with depression can develop coping mechanisms that, although they do not eliminate depression, could help to better manage physical pain, including headaches. In addition, some people with depression may experience a decrease in emotional reactivity, which could include a lower perception of pain. Symptom priority could be another possible explanation. If a person has depression, and if this is as dominant as the headaches, the latter, although present, may not be perceived with the same intensity. Likewise, pain desensitization processes could be acting. People with depression, in the presence of other symptoms, may become accustomed to certain levels of discomfort, which could make the headache be perceived as less significant compared to other more acute symptoms. On the other hand, it is possible that the anxiety associated with COVID-19 symptoms is so high that the headache is perceived as less important. In any case, further research is needed to understand the interaction between different psychosocial variables and emotional symptoms in predicting the maintenance of headaches.

One of the most novel aspects of our study is the analysis of the different variables of emotional symptomatology and personality and their relationship with the chronification of headaches. Our findings show a significant relationship between anxiety, burnout, and cognitive fusion. However, when entering these variables in the logistic regression model, the only variable that acts as a predictor of chronic headaches is stress, indicating that stress contributes to a 10.4% increase in the likelihood of chronic headaches. This is in line with many authors who claim that stress is closely linked to the development of headaches, stating that people who suffer high levels of stress are at risk of developing headaches [43,44]. However, the relationship between stress and chronic headaches has not been studied as much. The possible relationship between headache chronicity and different personality variables has not been studied either. Although there is scientific evidence that health professionals reported COVID symptomatology, headaches, and burnout during the COVID-19 pandemic, the relationship between them has not been studied so far [53]. A study by Katrin et al. (2017) relates stress as a potential prognostic factor for poor prognosis and unfavorable outcomes of preventive treatment in chronic headaches [54].

In our study, we proposed a moderate mediation model considering burnout as a variable consequence of the model, since burnout is a consequence associated with chronic stress [55]. This model proposes the chronification of stress (burnout) throughout the pandemic, starting from the anxiety suffered at the beginning of the pandemic, which led to a cognitive fusion in the second temporal moment, thus producing the development of burnout. Our study shows how chronic headaches can influence this model, observing that the fact of suffering chronic headaches increases burnout six times, two years after the start of the pandemic. In addition, our model suggests that people with chronic headaches do not experience this significant pattern. That is to say, they do not need to suffer anxiety and cognitive fusion to chronify stress, simply because suffering chronic headaches is a stressor to suffer burnout in the long term. The existing literature has studied the relationship between burnout and headaches in HCWs, stating that professionals who suffer burnout have a higher prevalence of headaches [44,56]. High levels of burnout have been reported among healthcare personnel in highly complex units such as intensive care units (ICUs) [57], leading to an increase in burnout-related symptoms such as headaches [5]. However, the novel relationship defined by our study, which states that chronic headaches can cause burnout, has not been studied so far.

It can be affirmed, from this point, that the presence of chronic headaches increases (by itself) the probability of suffering burnout. In people who do not have headaches, a direct relationship between anxiety and burnout is observed, and this relationship is clearly increased when the anxiety leads to cognitive fusion six months later.

### Research Limitations

This research has several limitations that should be considered. First, this study employed non-probabilistic convenience sampling, which may reduce the representativeness of the findings in relation to the broader population, as the sample was concentrated in specific communities. However, given the exceptional circumstances under which the study was conducted during the COVID-19 pandemic, this sampling approach was deemed necessary. The pandemic period imposed numerous challenges, including widespread confinement, elevated fear of contagion, and considerable difficulties in accessing healthcare professionals who were under substantial occupational strain and exhaustion. Due to these constraints, the research team decided to use convenience sampling to maximize the recruitment of HCWs and ensure that as many as possible could participate in the study. Importantly, despite this limitation, no significant differences were found in the variables of interest between the full initial participant group at the first time point (*n* = 1221) and the final study sample who completed all three time points (*n* = 259). This lack of significant discrepancies suggests that the potential bias introduced by convenience sampling may be minimal, supporting the robustness of the sample used in this study.

A second limitation is the absence of baseline data on whether HCWs had pre-existing headaches or anxiety symptoms before the onset of the pandemic. Without this information, it is challenging to ascertain the specific impact of pandemic-related stressors on these health outcomes, as individuals with pre-existing symptoms may have experienced exacerbated levels of anxiety and headaches during the pandemic. This limitation restricts our understanding of the unique effects of COVID-19 stressors on the mental health of HCWs who did not previously report these symptoms, potentially influencing this study’s findings on anxiety and headache prevalence.

Third, this study focuses primarily on psychological factors affecting the mental health of HCWs, with limited discussion of biological factors that may contribute to the persistence and chronification of symptoms such as headaches. Although biological influences were not the focus of this manuscript, we acknowledge their relevance to understanding these health outcomes. Therefore, we have emphasized their significance throughout the manuscript and underscore the importance of incorporating biopsychosocial models that integrate biological, psychological, and social factors in future research on these topics. This approach could yield a more comprehensive understanding of the factors contributing to healthcare workers’ mental health during and beyond the COVID-19 pandemic.

## 5. Conclusions

Despite the limitations of this research, it is essential to highlight the main contributions of the findings obtained, some of which are particularly innovative in comparison with previous studies. Among them, we highlight the protective role played by depression, when considered in interaction with other symptomatology variables, in the development of headaches at the beginning of the pandemic. This result is completely novel and may be explained by the fact that people with depression can develop coping mechanisms that, although they do not eliminate depression, could help to manage physical pain, including headaches. More research along these lines is needed.

Our article is in line with other authors in stating a direct association between elevated levels of stress and anxiety and the development of chronic headaches. However, the novel association is to state that the chronification of headaches is related to personality variables such as cognitive fusion. When we assess the predictor role of the variables as a whole, stress is the predictor variable, constituting a risk factor. With respect to our model, the presence of chronic headaches increases (by itself) the probability of suffering from burnout. In professionals who do not suffer from headaches, a direct relationship between anxiety and burnout is observed. The pattern is verified independently of gender and professional category.

In summary, it is crucial to address the psychological needs of HCWs to prevent and manage headaches in the work environment. Although many studies analyze the symptomatology related to the prevalence of headaches in health professionals, very few examine the role of variables that act as predictors in the development of headaches and their chronification.

## Figures and Tables

**Table 1:** Relationships of headaches (T1) with socio-demographic and occupational variables, emotional symptomatology, and psychosocial variables.

		Time 1 Headaches					
		Yes (*n* = 181)	No (*n* = 78)	Test		*p*	d/ V *(p)*
Gender	Woman (*n* = 211)	152 (72%)	59 (28%)	*X* ^2^	2.509	0.113	0.098 (0.11)
	Man (*n* = 48)	29 (60.4%)	19 (39.6%)				
Age	44.36 (DE = 9.84)	42.66 (9.55)	46.05 (9.97)	t	2.579	0.010	0.35
Cohabitation as a couple	YES (*n* = 182)	129 (70.9%)	53 (29.1%)	*X* ^2^	2.177	0.337	0.092 (0.33)
	NO (*n* = 77)	52 (67.5%)	25 (32.5%)				
Professional category	Physician (*n* = 65)	38 (58.5%)	27 (41.5%)	*X* ^2^	5.788	0.049	0.163 (0.22)
	Nurse (*n* = 151)	113 (74.8%)	38 (25.2%)				
	Technician (*n* = 43)	30 (69.7%)	13 (30.3%)				
Service	ICU (*n* = 93)	66 (71%)	27 (29%)	*X* ^2^	17.786	0.023	0.26 (0.02)
	COVID Hospitalization (*n* = 64)	51 (79.7%)	13 (20.3%)				
	Primary Care (*n* = 44)	30 (68.2%)	14 (31.8%)				
	Emergency (*n* = 34)	25 (73.5%)	9 (26.5%)				
	Others (*n* = 24)	8 (33.3%)	16 (66.6%)				
Professional experience	11.06 (SD = 9.29)	10.16 (8.86)	11.84 (9.91)	t	1.355	0.177	0.018
Transfer to the ICU due to the pandemic	YES (*n* = 43)	28 (65.1%)	15 (34.9%)	*X* ^2^	0.557	0.456	0.046 (0.45)
	NO (*n* = 216)	153 (70.8%)	63 (29.2%)				
Availability of PPE	YES (*n* = 108)	68 (63%)	40 (37%)	*X* ^2^	4.216	0.040	0.128 (0.04)
	NO (*n* = 151)	113 (74.8%)	38 (25.2%)				
Previous chronic illness	YES (*n* = 68)	46 (67.6%)	22 (32.4%)	*X* ^2^	0.219	0.640	0.029 (0.64)
	NO (*n* = 191)	135 (70.7%)	56 (29.3%)				
Symptomatology COVID		4.26 (2.64)	1.23 (1.61)	t	−9.41	<0.001	1.38
Worried about own contagion		2.80 (0.90)	2.59 (0.95)	t	−1.649	0.100	0.23
Worried about family contagion		3.67 (0.73)	3.22 (0.97)	t	−4.127	<0.001	0.53
Anxiety		6.35 (4.91)	4.17 (3.83)	t	−3.473	<0.001	0.50
Depression		6.07 (4.62)	4.84 (3.89)	t	−2.059	0.029	0.29
Stress		10.49 (4.75)	8.42 (4.67)	t	−3.237	0.001	0.44
Insomnia		12.90 (5.77)	9.94 (5.99)	t	−3.740	<0.001	0.50
Social support (MSPSS)	TOTAL	5.81(1.14)	5.69 (1.33)	t	−0.679	0.497	0.10
	Family	5.90 (1.20)	5.81 (1.18)	t	−0.511	0.609	0.08
	Friends	5.66 (1.33)	5.60 (1.53)	t	−0.313	0.755	0.04
Self-efficacy (GSES)		29.12 (3.96)	29.32 (4.32)	t	0.360	0.719	0.05
Resilience (RS-14)		78.28 (13.6)	78.66 (15.6)	t	0.199	0.843	0.02

t: Student’s *t*-test; *X^2^*: Pearson’s chi-square; d: Cohen’s d; V: Cramer’s V.

**Table 2:** Headache and chronic headache.

	T1	T2	T1 and T2 (Chronic Headache)
Yes	181 (69.9%)	191 (73.7%)	154 (59.5%)
No	78 (30.1%)	68 (26.3%)	105 (40.5%)

T1: period between 5 May and 21 June 2020, T2: period between January–April 2021.

**Table 3:** Logistic regression for headache prediction (only variables statistically significant at the bivariate level (T1)) was taken into account.

	Coefficient (B)	SE	Wald	*p*	Exp (B) (Odds Ratio)
Nurse	0.200	0.514	0.151	0.697	1.221
Hospitalization	0.105	0.402	0.079	0.798	0.812
Emergency	0.098	0.345	0.076	0.802	0.768
ICU	−0.129	0.454	0.081	0.776	0.879
PPE	0.301	0.419	0.517	0.472	1.351
Age	−0.007	0.022	0.097	0.756	0.993
COVID-19 symptoms	1.113	0.187	35.420	<0.001	3.042
Worried about family contagion	0.250	0.233	1.152	0.283	1.284
Anxiety	0.128	0.076	2.830	0.093	1.136
Depression	−0.241	0.094	6.641	0.010	0.786
Stress	0.045	0.076	0.349	0.555	1.046
Insomnia	0.009	0.047	0.037	0.848	1.009

B: Beta coefficient, SE: Standard error, Exp (B): Exponentiated Coefficient.

**Table 4:** Relationships of chronic headaches with emotional symptomatology and personality.

		Time 1 and 2 Chronic Headaches				
		Yes (*n* = 154)	No (*n* = 105)	t	*p*	d
Anxiety T2		5.56 (4.37)	4.34 (4.78)	−2.123	0.035	0.27
Depression T2		6.07 (4.36)	5.17 (4.58)	−1.595	0.112	0.20
Stress T2		10.44 (4.38)	8.39 (5.21)	−3.428	0.001	0.43
Insomnia T2		10.56 (6.20)	9.68 (6.54)	−3.740	0.275	0.14
Social support (MSPSS)	Total	5.81(1.10)	5.72 (1.34)	−0.679	0.544	0.07
	Family	5.87 (1.18)	5.86 (1.22)	−0.071	0.943	<0.01
	Friends	5.67 (1.25)	5.60 (1.58)	−0.395	0.693	0.05
Self-efficacy		29.01 (3.92)	29.41 (4.27)	0.775	0.439	0.09
Resilience		78.49 (12.5)	78.25 (16.5)	−0.131	0.896	0.02
Cognitive fusion		23.31 (10.8)	19.9 (10.4)	−2.494	0.013	0.32
Burnout		71.37 (16.2)	65.84 (20.7)	−2.399	0.017	0.30

t: Student’s *t*-test; d: Cohen’s d.

**Table 5:** Logistic regression for chronic headache prediction (only variables statistically significant at the bivariate level (T1) was taken into account.

	Coefficient (B)	SE	Wald	*p*	Exp (B) (Odds Ratio)
Anxiety	−0.029	0.045	0.414	0.520	0.971
Stress	0.099	0.043	5.284	0.022	1.104
Cognitive fusion	0.009	0.017	0.300	0.584	1.009

**Table 6:** Moderate mediation model: Regression of anxiety and cognitive fusion (mediator) on burnout with chronic headache as moderator (*n* = 259).

Regression of Anxiety and Cognitive Fusion (Mediator) on Burnout with Chronic Headache as a Moderator				
Outcome Variable: Cognitive Fusion				
	B (SE)	t	*p*	[LLCI-ULCI]
X: Anxiety (A)	1.17(.12)	9.59	<0.001	[0.934/1.41]
Age (covariate)	0.029 (.07)	0.424	0.671	[−0.011/0.16]
Nurse (covariate)	−0.82(1.43)	−0.576	0.564	[−3.64/1.99]
Model summary	R = 0.51; R^2^ = 0.26; F = 92.12; *p* < 0.001			
Outcome variable: Burnout				
	B (SE)	t	*p*	[LLCI-ULCI]
X: Anxiety (A)	1.25(.39)	3.20	0.001	[0.481/2.02]
M: Cognitive fusion (CF)	0.77(.11)	7.19	<0.001	[0.56/0.98]
Mo: Chr. headache (CH)	6.4 (3.17)	2.03	0.043	[0.20/12.72]
Int A CH	−0.78 (0.45)	−1.75	0.048	[−1.69/−0.09]
Age (covariate)	−0.01(0.11)	−0.054	0.956	[−0.22/0.21]
Nurse (covariate)	−0.22(2.25)	−0.100	0.920	[−4.67/4.21]
Model summary	R = 0.60; R^2^ = 0.36; F = 19.84; *p* < 0.001			
Conditional effects of Predictor (A) at values of Moderator (CH)				
CH value	Eff (SE)	t	*p*	[LLCI-ULCI]
0	1.25 (0.39)	3.20	0.001	[0.481/2.02]
1	0.45 (0.30)	1.48	0.139	[−0.14/1.05]
Direct and indirect effects of Anxiety on Burnout				
Conditional direct effects of Anxiety on Burnout				
CH value	Eff (SE)	t	*p*	[LLCI-ULCI]
0	1.25 (0.39)	2.20	0.001	[0.481/2.02]
1	0.45 (0.30)	1.48	0.139	[−0.14/1.05]
Indirect effect of Anxiety on Burnout				
	Effect (BootSE)		[BootLLCI, BootULCI]	
Cognitive fusion	0.937(0.151)		[0.66/1.25]	

## Data Availability

Research data will be available upon request to the corresponding author.

## References

[1] Xie W., Li R., He M., Cui F., Sun T., Xiong J., Zhao D., Na W., Liu R., Yu S. (2020). Prevalence and Risk Factors Associated with Headache amongst Medical Staff in South China. J. Headache Pain.

[2] Saylor D., Steiner T.J. (2018). The Global Burden of Headache. Semin. Neurol..

[3] Onwuekwe I., Onyeka T., Aguwa E., Ezeala-Adikaibe B., Ekenze O., Onuora E. (2014). Headache Prevalence and Its Characterization amongst Hospital Workers in Enugu, South East Nigeria. Head Face Med..

[4] Goadsby P.J., Holland P.R., Martins-Oliveira M., Hoffmann J., Schankin C., Akerman S. (2017). Pathophysiology of Migraine: A Disorder of Sensory Processing. Physiol. Rev..

[5] Carvalho D.P., de Rocha L.P., Pinho E.C., de Tomaschewski-Barlem J.G., Barlem E.L.D., Goulart L.S. (2019). Workloads and Burnout of Nursing Workers. Rev. Bras. Enferm..

[6] Leyva-Vela B., Jesús Llorente-Cantarero F., Henarejos-Alarcón S., Martínez-Rodríguez A. (2018). Psychosocial and Physiological Risks of Shift Work in Nurses: A Cross-Sectional Study. Cent. Eur. J. Public Health.

[7] Healy D., Minors D.S., Waterhouse J.M. (1993). Shiftwork, Helplessness and Depression. J. Affect. Disord..

[8] Brandes J.L. (2012). Migraine in Women. Continuum.

[9] Pavlović J.M. (2021). Headache in Women. Continuum.

[10] Farnell S., Dawson D. (2006). “It’s Not like the Wards”. Experiences of Nurses New to Critical Care: A Qualitative Study. Int. J. Nurs. Stud..

[11] Magro-Morillo A., Boulayoune-Zaagougui S., Cantón-Habas V., Molina-Luque R., Hernández-Ascanio J., Ventura-Puertos P.E. (2020). Emotional Universe of Intensive Care Unit Nurses from Spain and the United Kingdom: A Hermeneutic Approach. Intensive Crit. Care Nurs..

[12] Zarei N., Negarandeh R., Eghbali M. (2024). Prevalence of Headaches in Healthcare Workers While Using Personal Protective Equipment during the COVID-19 Pandemic: A Systematic Review and Meta-Analysis. BMJ Open.

[13] Etgu F., Onder S. (2021). Skin Problems Related to Personal Protective Equipment among Healthcare Workers during the COVID-19 Pandemic (Online Research). Cutan. Ocul. Toxicol..

[14] Jafari E., Togha M., Kazemizadeh H., Haghighi S., Nasergivehchi S., Saatchi M., Ariyanfar S. (2021). Evaluation of Headache Associated with Personal Protective Equipment during COVID-19. Brain Behav..

[15] Nasergivehchi S., Togha M., Jafari E., Sheikhvatan M., Shahamati D. (2023). Headache Following Vaccination against COVID-19 among Healthcare Workers with a History of COVID-19 Infection: A Cross-Sectional Study in Iran with a Meta-Analytic Review of the Literature. Head Face Med..

[16] Ekizoglu E., Gezegen H., Yalınay Dikmen P., Orhan E.K., Ertaş M., Baykan B. (2022). The Characteristics of COVID-19 Vaccine-Related Headache: Clues Gathered from the Healthcare Personnel in the Pandemic. Cephalalgia.

[17] Cho S.J., Chu M.K. (2015). Risk Factors of Chronic Daily Headache or Chronic Migraine. Curr. Pain Headache Rep..

[18] Watson N.F., Badr M.S., Belenky G., Bliwise D.L., Buxton O.M., Buysse D., Dinges D.F., Gangwisch J., Grandner M.A., Kushida C. (2015). Recommended Amount of Sleep for a Healthy Adult: A Joint Consensus Statement of the American Academy of Sleep Medicine and Sleep Research Society. J. Clin. Sleep Med..

[19] Benkli B., Kim S.Y., Koike N., Han C., Tran C.K., Silva E., Yan Y., Yagita K., Chen Z., Yoo S.-H. (2023). Circadian Features of Cluster Headache and Migraine: A Systematic Review, Meta-Analysis, and Genetic Analysis. Neurology.

[20] Trusso Sfrazzetto G., Santonocito R. (2022). Nanomaterials for Cortisol Sensing. Nanomaterials.

[21] Peres M.F.P., Mercante J.P.P., Tobo P.R., Kamei H., Bigal M.E. (2017). Anxiety and Depression Symptoms and Migraine: A Symptom-Based Approach Research. J. Headache Pain.

[22] Dueland A.N., Leira R., Burke T.A., Hillyer E.V., Bolge S. (2004). The Impact of Migraine on Work, Family, and Leisure among Young Women—A Multinational Study. Curr. Med. Res. Opin..

[23] Linde M., Dahlöf C. (2004). Attitudes and Burden of Disease among Self-Considered Migraineurs—A Nation-Wide Population-Based Survey in Sweden. Cephalalgia.

[24] Berg J. (2004). Economic Evidence in Migraine and Other Headaches: A Review. Eur. J. Health Econ..

[25] Leonardi M., Steiner T.J., Scher A.T., Lipton R.B. (2005). The Global Burden of Migraine: Measuring Disability in Headache Disorders with WHO’s Classification of Functioning, Disability and Health (ICF). J. Headache Pain.

[26] Schoemann A.M., Boulton A.J., Short S.D. (2017). Determining Power and Sample Size for Simple and Complex Mediation Models. Soc. Psychol. Personal. Sci..

[27] Cobo-Cuenca A.I., Fernández-Fernández B., Carmona-Torres J.M., Pozuelo-Carrascosa D.P., Laredo-Aguilera J.A., Romero-Gómez B., Rodríguez-Cañamero S., Barroso-Corroto E., Santacruz-Salas E. (2022). Longitudinal Study of the Mental Health, Resilience, and Post-Traumatic Stress of Senior Nursing Students to Nursing Graduates during the COVID-19 Pandemic. Int. J. Environ. Res. Public Health.

[28] Jubin J., Delmas P., Gilles I., Oulevey Bachmann A., Ortoleva Bucher C. (2023). Factors Protecting Swiss Nurses’ Health during the COVID-19 Pandemic: A Longitudinal Study. BMC Nurs..

[29] Ruiz F.J., Martín M.B.G., Falcón J.C.S., González P. (2017). O The Hierarchical Factor Structure of the Spanish Version of Depression Anxiety and Stress Scale-21. Int. J. Psychol. Psychol. Ther..

[30] Henry J., Crawford J. (2005). The Short-Form Version of the Depression Anxiety Stress Scales (DASS-21): Construct Validity and Normative Data in a Large Non-Clinical Sample. Br. J. Clin. Psychol..

[31] Fabbri M., Beracci A., Martoni M., Meneo D., Tonetti L., Natale V. (2021). Measuring Subjective Sleep Quality: A Review. Int. J. Environ. Res. Public Health.

[32] Bastien C.H., Vallières A., Morin C.M. (2001). Validation of the Insomnia Severity Index as an Outcome Measure for Insomnia Research. Sleep Med..

[33] Zimet G.D., Powell S.S., Farley G.K., Werkman S., Berkoff K.A. (1990). Psychometric Characteristics of the Multidimensional Scale of Perceived Social Support. J. Pers. Assess..

[34] Calderón C., Ferrando P.J., Lorenzo-Seva U., Gómez-Sánchez D., Fernández-Montes A., Palacín-Lois M., Antoñanzas-Basa M., Rogado J., Manzano-Fernández A., Ferreira E. (2021). Multidimensional Scale of Perceived Social Support (MSPSS) in Cancer Patients: Psychometric Properties and Measurement Invariance. Psicothema.

[35] De Las Cuevas C., Peñate W. (2015). Validation of the General Self-Efficacy Scale in Psychiatric Outpatient Care. Psicothema.

[36] Suárez P.S., García A.M.P., Moreno J.B. (2000). Moreno Escala de Autoeficacia General: Datos Psicométricos de La Adaptación Para Población Española. Psicothema.

[37] Sánchez-Teruel D., Robles-Bello M.A. (2015). Escala de Resiliencia 14 Ítems (RS-14): Propiedades Psicométricas de La Versión En Español. [14-Item Resilience Scale (RS)-14): Psychometric Properties of the Spanish Version.]. Rev. Iberoam. Diagnóstico Evaluación Psicol..

[38] Gillanders D.T., Bolderston H., Bond F.W., Dempster M., Flaxman P.E., Campbell L., Kerr S., Tansey L., Noel P., Ferenbach C. (2014). The Development and Initial Validation of the Cognitive Fusion Questionnaire. Behav. Ther..

[39] Romero-Moreno P., Márquez-González M., Losada A., Gillanders D., Fernández-Fernández V. (2014). Cognitive Fusion in Dementia Caregiving: Psychometric Properties of the Spanish Version of the “Cognitive Fusion Questionnaire”. Behav. Psychol..

[40] Maslach C., Jackson S., Leiter M., Zalaquett C.P., Wood R.J. (1997). The Maslach Burnout Inventory Manual. Evaluating Stress: A Book of Resources.

[41] Gil-Monte P.R. (2005). Factorial Validity of the Maslach Burnout Inventory (MBI-HSS) among Spanish Professionals. Rev. Saude Publica.

[42] Sahebi A., Hasheminejad N., Shohani M., Yousefi A., Tahernejad S., Tahernejad A. (2022). Personal Protective Equipment-Associated Headaches in Health Care Workers during COVID-19: A Systematic Review and Meta-Analysis. Front. Public Health.

[43] Lin K.-C., Huang C.-C., Wu C.-C. (2007). Association between Stress at Work and Primary Headache among Nursing Staff in Taiwan. Headache.

[44] Nadaoka T., Kanda H., Oiji A., Morioka Y., Kashiwakura M., Totsuka S. (1997). Headache and Stress in a Group of Nurses and Government Administrators in Japan. Headache.

[45] García-Hedrera F.J., Gil-Almagro F., Carmona-Monge F.J., Peñacoba-Puente C., Catalá-Mesón P., Velasco-Furlong L. (2021). Intensive Care Unit Professionals during the COVID-19 Pandemic in Spain: Social and Work-Related Variables, COVID-19 Symptoms, Worries, and Generalized Anxiety Levels. Acute Crit. Care.

[46] Olesen J., Gustavsson A., Svensson M., Wittchen H.-U., Jönsson B. (2012). The Economic Cost of Brain Disorders in Europe. Eur. J. Neurol..

[47] Ong J.J.Y., Bharatendu C., Goh Y., Tang J.Z.Y., Sooi K.W.X., Tan Y.L., Tan B.Y.Q., Teoh H.-L., Ong S.T., Allen D.M. (2020). Headaches Associated with Personal Protective Equipment—A Cross-Sectional Study Among Frontline Healthcare Workers During COVID-19. Headache.

[48] Gil-Almagro F., Carmona-Monge F.J., García-Hedrera F.J., Peñacoba-Puente C. (2024). Headache and Associated Psychological Variables in Intensive Care Unit Nurses during the COVID-19 Pandemic: A Prospective Study. J. Clin. Med..

[49] Martelletti P., Bentivegna E., Spuntarelli V., Luciani M. (2021). Long-COVID Headache. SN Compr. Clin. Med..

[50] Chwalisz B.K., Le V.K., Cheng J.R., Jain A., Brandon Westover M., Cheng H.T. (2023). COVID-19-Induced Headache in Boston and the Vicinity. J. Clin. Virol. Plus.

[51] Wachowska K., Bliźniewska-Kowalska K., Sławek J., Adamczyk-Sowa M., Szulc A., Maes M., Kuan-Pin S., Gałecki P. (2023). Common Pathomechanism of Migraine and Depression. Psychiatr. Pol..

[52] Viudez-Martínez A., Torregrosa A.B., Navarrete F., García-Gutiérrez M.S. (2024). Understanding the Biological Relationship between Migraine and Depression. Biomolecules.

[53] Mahlangu P., Sikweyiya Y., Gibbs A., Shai N., Machisa M. (2023). “I Carry the Trauma and Can Vividly Remember”: Mental Health Impacts of the COVID-19 Pandemic on Frontline Health Care Workers in South Africa. Int. J. Environ. Res. Public Health.

[54] Probyn K., Bowers H., Caldwell F., Mistry D., Underwood M., Matharu M., Pincus T. (2017). Prognostic Factors for Chronic Headache: A Systematic Review. Neurology.

[55] Edú-Valsania S., Laguía A., Moriano J.A. (2022). Burnout: A Review of Theory and Measurement. Int. J. Environ. Res. Public Health.

[56] Salvagioni D.A.J., Melanda F.N., Mesas A.E., González A.D., Gabani F.L., Andrade S.M. (2017). Physical, Psychological and Occupational Consequences of Job Burnout: A Systematic Review of Prospective Studies. PLoS ONE.

[57] Keane A., Ducette J., Adler D.C. (1985). Stress in ICU and Non-ICU Nurses. Nurs. Res..

